# Reading Photography in French Nineteenth Century Journals

**DOI:** 10.1080/13688804.2018.1530974

**Published:** 2018-10-09

**Authors:** Beatriz Pichel

**Affiliations:** Photographic History Research Centre, De Montfort University, Clephan Building, 1.01d.Leicester, LE1 9BH, United Kingdom of Great Britain and Northern Ireland.

**Keywords:** photography, medical journals, theatre, nineteenth century, France

## Abstract

This article explores how photographs published in the French medical and, to some extent, the popular press helped readers to interpret expressions and gestures as signs of emotional states, morbid conditions and physiological and psychological processes. The first two sections examine the use of photography to visualise normal and pathological bodies through measurements and experiments in the medical press, particularly Nouvelle Iconographie de la Salpêtrière, Archives de Neurologie and L’Année Psychologique. The next two sections study how the development of new photographic processes such as the magnesium flash and chronophotography created new conditions in which the body could be visually scrutinised in the medical press as well as popular journals such as Le Théâtre and the general scientific journal La Nature. This analys results in two main findings: 1) medical journals used photography to assert their own disciplinary identities, and 2) photography acted as a potential bridge between audiences, as some medical and popular journals shared the same beliefs regarding photography’s ability to represent the human body, but approached photographic innovations from different, albeit complementary, ways.

Reading the body has always been a key issue in Western medicine, and it became a particularly important skill by the end of the nineteenth century in France.^1^ The physiognomic tradition, which associated facial traits to personal characteristics, entered a new phase with the studies on the correlation between facial expressions and emotional states carried out by Duchenne de Boulogne and Darwin in the 1860s and 1870s.^2^ During the same years, Alphonse Bertillon applied similar principles to his work at the Police Prefecture in Paris, creating identification systems through the examination of the morphology of the face and other singular marks and the use of photographic portraits.^3^ Finally, Jean–Martin Charcot and his team at the Parisian hospital La Salpêtrière developed the anatomo-clinical method based on the visual observation of the body, linking visible symptoms with anatomical and neurological lesions.^4^ The bodily language of Charcot’s hysterical patients even penetrated into the public sphere as actresses copied some of their most famous gestures.^5^ In *fin-de-siècle* France, the body on display became a means to accessing the emotional, psychological and pathological internal processes that scientists and the public aimed to understand.

This article focuses on two factors that contributed to the shaping and development of this discussion in the late nineteenth century. Firstly, the multiplication of scientific journals and contributors had turned the medical press into a key site where theories were tested, discussed and (eventually) consolidated.^6^ Secondly, photography had become a powerful tool in science. Although the objectivity of its images was always challenged, the ability of photography to freeze the subject and to reproduce the images in multiple formats proved very useful in medical and popular contexts.^7^ As Geoffrey Belknap has demonstrated, both factors were intertwined.^8^ Photographs in the press were not mere illustrations, but had an impact on how science was communicated and understood. Equally, photographs became specific scientific objects when they were published and discussed in journals.^9^

In the following pages, I explore how photographs published in the medical and, to some extent, the popular press helped readers to interpret expressions and gestures as signs of emotional states, morbid conditions and physiological and psychological processes. The first two sections examine the use of photography to visualise normal and pathological bodies through measurements and experiments in the medical press, particularly *Nouvelle Iconographie de la Salpêtrière* (1888–1918, *Nouvelle* hereafter), *Archives de Neurologie* (1870–1907, *Archives* hereafter) and *L’Année Psychologique* (1895 to the present day, *L’Année* hereafter). The next two sections move from the analysis of images to the analysis of technologies, examining how the development of new photographic processes such as the magnesium flash and chronophotography created new conditions in which the body could be visually scrutinised. Taking up Belknap’s analysis, these sections consider photography as both a visual tool and a topic of discussion in the medical press as well as popular journals such as *Le Théâtre* (1898–1912) and the general scientific journal *La Nature* (1873–1960).^10^

This investigation applies the concepts and methods of photographic history to the history of the medical press, resulting in two main findings.^11^ Firstly, medical journals used photography to assert their own disciplinary identities. As the next sections show, different approaches to psychology and physiology materialised in journals which granted different roles to photography. Secondly, photography acted as a potential bridge between audiences, as some medical and popular journals shared the same beliefs regarding photography’s ability to represent the human body, but approached photographic innovations such as artificial lighting from different, albeit complementary ways. In conclusion, tracing photography in medical journals contributes to a better understanding of the medical press as a written visual media, and offers elements of analysis for the study of how related disciplinary fields developed their own identities.

## Visualising Bodily Measurements

The first issue of *Nouvelle* opened with a description of its aims. In the spirit of its predecessor *Iconographie photographique de la Salpêtrière* (1875–1880), *Nouvelle* intended to ‘make use of the numerous figurative documents that accumulate daily in the Salpêtrière’s albums.’^12^ The foreword emphasised the value of visual documents, affirming that they ‘complete the written observation, bring old cases back to life and facilitate the comparison of analogue cases even when the patient is no longer here.’^13^ It continued, ‘furthermore, this publication will allow those interested in neurological disorders to judge for themselves.’^14^ Photography, therefore, was important not just because it gave ‘exact representations’ of patients.^15^ Doctors at the clinic of nervous diseases at the Salpêtrière valued the taking of the images as much as sharing them with others who might find them useful. Photographs were intended to open conversations between specialists, and medical journals were the best place to achieve this goal.

*Nouvelle* situated itself in a particular tradition when its foreword mentioned *Iconographie* and *Archives* as its predecessors. The three journals had been founded or directed by Charcot and therefore shared an interest in neuropathology. Désiré Magloire Bourneville, a key figure in the creation of *Iconographie* and doctor and former intern at the Salpêtrière, became editor-in-chief and a prominent contributor to *Archives* when he joined the Bicêtre hospital in Paris.^16^ In his articles, Bourneville incorporated visual evidence, particularly photographs. The content of these images, and especially the role of the photographs in the articles, created a continuity between *Iconographie*, *Archives* and *Nouvelle*. Visualising the body in photographs taken at hospital came to define the discipline’s approach to nervous disorders, and this shared visual culture became the identity mark of the three journals.

**FIGURE 1.**
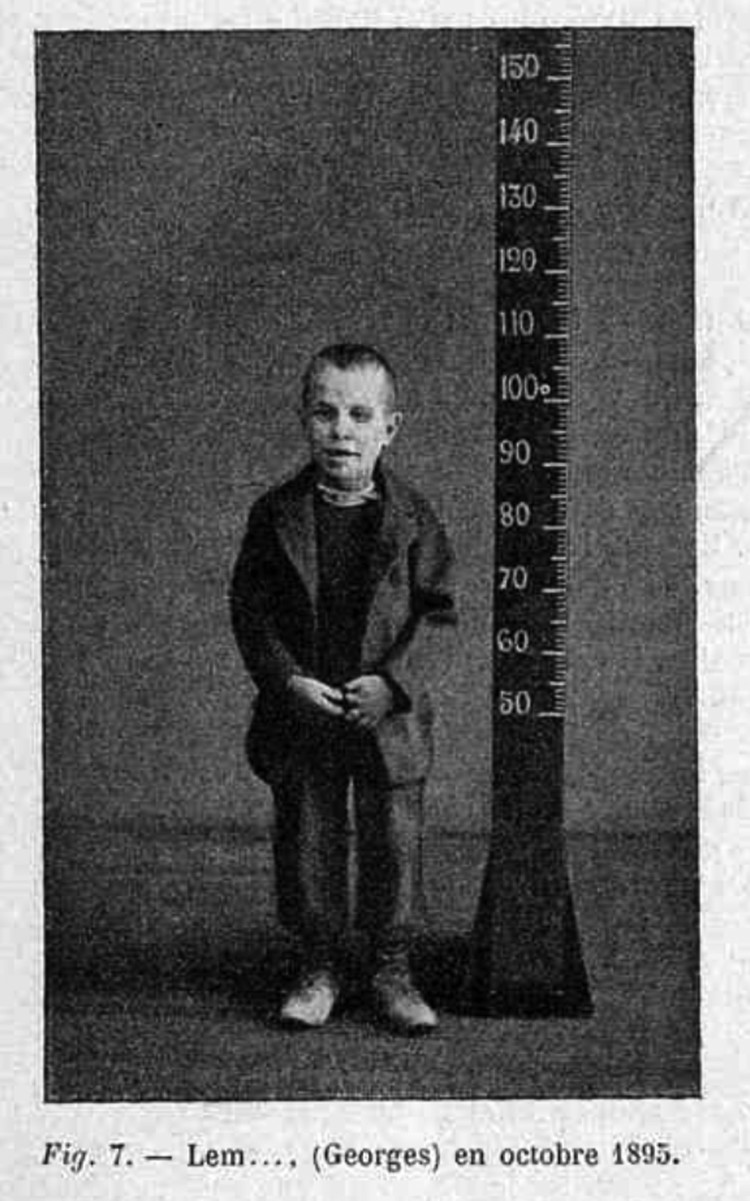
‘Lem … (Georges) en Octobre 1895’, Archives de Neurologie, 16 No. 91 (1903), Jubilotheque

One of the ways in which photographs became useful tools in the visualisation of nervous disorders was through bodily measurements. The articles on idiocy written by Bourneville for *Archives* are good examples. The first of them discussed the case of a child affected of ‘severe idiocy, dwarfism and infantilism’, and included eight images (Figure 1).^17^ All of them corresponded to the same patient, who had been photographed every one or two years since his arrival in 1890.^18^ The photographs always followed the same conventions: the child was at the centre of the image, standing up against a dark grey background. Next to him, a scale indicated to the viewer, either the doctor or the journal’s reader, the height of the patient. The child was clothed, staring at the camera. Only on one occasion, in 1894 and aged 7, did the patient pose touching the scale and smiling.^19^

All the photographs that accompanied Bourneville’s articles presented these characteristics, and two articles published in *Nouvelle* also compared pathological bodies to a scale.^20^ The scale, therefore, had a key role in these journals. Firstly, measuring the body through its comparison to scales linked these images to anthropometric studies such as those carried out by Bertillon, which aimed to quantify the body shape.^21^ As Christopher Pinney has argued, anthropometry sought to ‘transform the presence of unique bodies into what we might think of as somatic prototypes.’^22^ In Bourneville’s photographs, the scale displayed visually the individuality of the subject at the same time that categorised it as belonging to a particular group; the ‘idiots’.^23^ Secondly, the scale brought all the photographs together. Regardless of the particular condition that needed to be highlighted, the scale remained stable, facilitating the comparison among the bodies. Moreover, all the images published in Bourneville’s articles were of the same size, and occupied the same place in the page, which reinforced their continuity not only between the photographs belonging to the same article, but between articles over time. The role of the photographs in the articles also remained the same. Photographic images were part of clinical histories, which systematically described the development of the children’s bodies and their conditions. The photographs expressed by visual means the same content that was described in the texts.^24^ A key element of this repetition was the scale, which provided visual evidence of the figures discussed in the main text.

**FIGURE 2.**
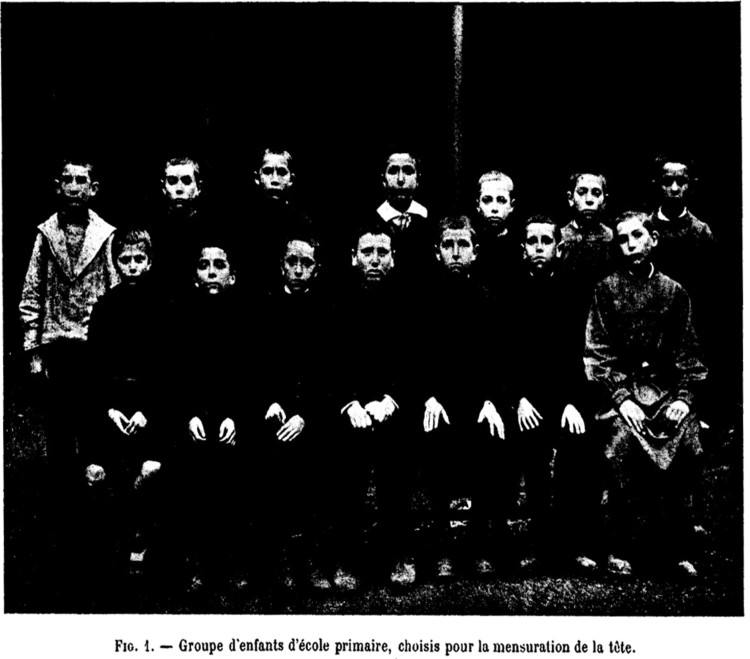
‘Groupe d’enfants d’école primaire, choisis pour la mensuration de la tête’, L’anné psychologique, 7 (1901): 357–402. Free of copyright

The visual measurement of the body by means of the standardisation of photographic images became a convention in *Archives* and *Nouvelle*, and contributed to the consideration of these photographs as authoritative medical objects. However, other disciplines followed different strategies. In 1901, *L’Année* published psychologist Alfred Binet’s research on the relation between the measurements of the head and the intelligence of children aged between 11 and 13.^25^ In the article, Binet included tables, statistics and one photograph: a portrait of a group of fourteen students at a school in Seine-et-Marne (Figure 2).^26^ Seven were sitting on a bench, with their hands on their knees, while the rest stood behind them. They were all wearing school clothes, and stared fixedly at the camera. The wall behind the children suggests that the photograph had been taken outdoors. The fourteen children had been selected out of one hundred due to the different measurements of their heads. Half of them had been classified as ‘intelligent’, while the other half had been deemed ‘unintelligent’. According to Binet,

By examining these 14 physiognomies, the reader will realise the kind of individual differences that I have found in my subjects. I invite the reader to guess who are, in the group, the most intelligent subjects. This judgement will benefit from the comparison with other physiognomies, while it will encounter more difficulties due to the immobility of the heads and their conventional expression – the living being always more evocative than the photographic portrait.^27^

This paragraph is the only reference to the photograph in the whole article, which went on to discuss calculations on seriation and the average of each of the measurements. In the article, Binet compared numerical figures in tables, which helped to make sense of the correlation between the measurements of the head and the children’s intelligence. The photograph, however, compared children to children. The image was not about measurements, but about sizes. The reader could only perceive whose head was bigger, and whose was smaller. This visual exercise was not intended to display the measurements of body parts, but to demonstrate how difficult it was to evaluate the intelligence of children through photographic documents.^28^ As the above-mentioned paragraph noted, photographs froze the expression of the children, leaving aside the most interesting nuances of their physiognomy, which contributed to the reader’s inability to assess their intelligence. Therefore, while *Archives* presented photographs as a visual aid that reinforced Bourneville’s theories, *L’Année* denied that photographs were useful in interpreting bodily measurements.

The visualisation of the children’s bodies was nonetheless important. Binet had started his career as an intern in Charcot’s service, but he had abandoned the pathological method when he left the Salpêtrière. As head of the Psychology laboratory at the Sorbonne, Binet favoured normal psychology.^29^ Accordingly, he photographed children in schools, not in hospitals.^30^ Images like Figure 2 mixed intelligent and unintelligent children, stressing the continuity among them. The function of this photograph in the article, therefore, was not to simply visualise the different sizes of children’s heads. The publication of such an image in *L’Année* stated the journal’s point of view in relation to psychological research. While the photograph did not have specific scientific value (it was not evidence of Binet’s theory), it helped to create the identity of the journal and the kind of psychology they supported.

## Photography and Medical Experiments

Most of the photographs in *L’Année* were linked to experiments. This is the case for Binet’s ‘Causerie pédagogique’ (1907), which included two photographs of his studies on the breathing of children when they were writing.^31^ The images showed the same child in two different postures: the ‘ideal attitude’, characterised by the straight back and the separation of the chest from the table, and the bad attitude, in which the upper body leaned to the front and to the right, and the table compressed the chest.^32^ The reader could also see on the table the child’s notebook and Marey’s pneumograph, which registered the breathing of the child. The two images, which were included to support the conclusions of the research, were compared to the traces of nasal breathing taken by a pneumograph (visible in the photograph).^33^ The graph demonstrated that the position of the child’s body did not affect the breathing, contrary to previous research.^34^

The photographs published in *L’Année* were superfluous from a scientific point of view. The images did not play any role in the experiment itself or intervene in the results, but only attested to the fact that the experiment had been performed. These two photographs in particular were misleading, as the compressed chest in the ‘bad attitude’ photograph might suggest breathing changes. In spite of this, the photographs were important from the point of view of the journal’s identity. Firstly, they showed students in a normal environment (not the clinic), focusing again on the normal aspects of psychology. Secondly, they revealed the use of physiological instruments such as Marey’s penumograph. Then, the focus of the image was not on the child’s body but on the laboratory instrument, which had become a symbol of Binet’s experimental psychology.^35^

**FIGURE 3.**
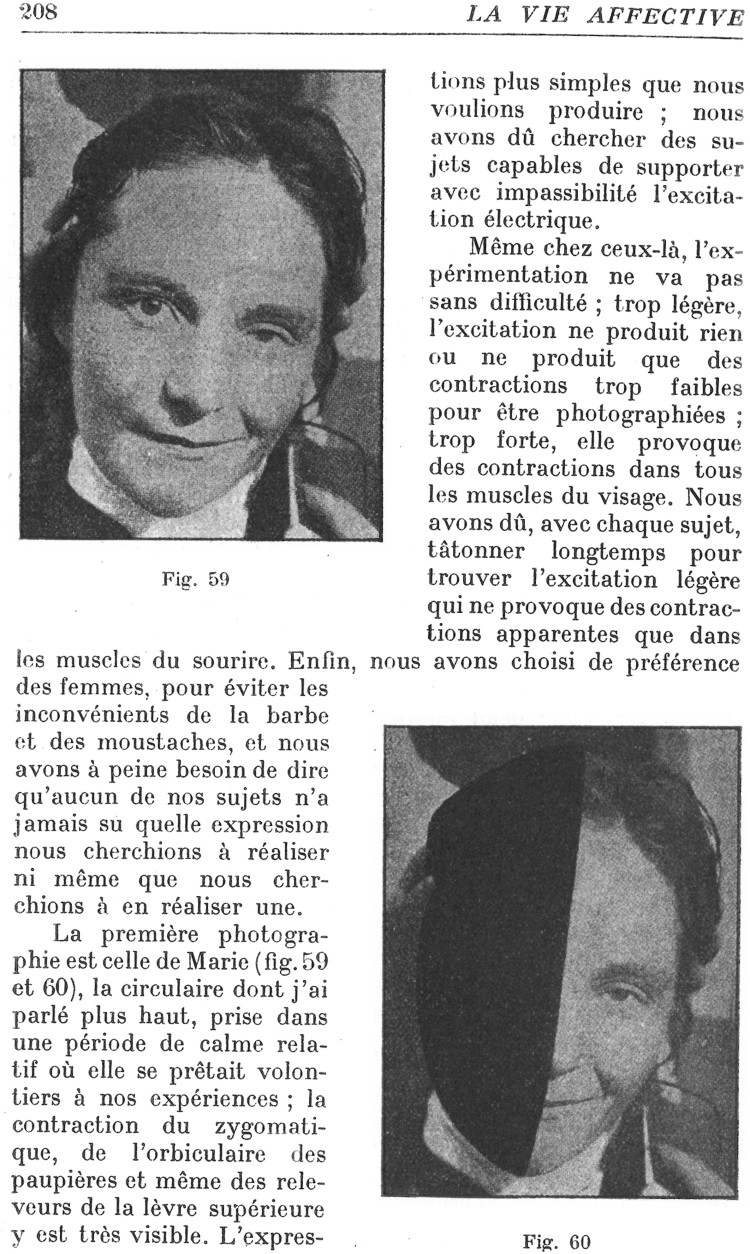
‘Figure 6- Marie’ Le sourire, Georges Dumas. Reprinted in Dumas, La Vie Affective. Physiologie - Psychologie - Socialisation (Paris: Presses Universitaires de France, 1948), 208.

Contrary to the approach followed by *L’Année*, other journals attributed a greater role to photography. As *Nouvelle*’s foreword stated, the publication of photographs sought to create a dialogue in the medical community, offering the images for discussion. In the case of experiments, this was particularly important, as photographs made the readers participants in the interpretation of the results. *Nouvelle* and other journals related to Charcot’s school, such as *Revue philosophique de la France et l’étranger* (1876 to the present day, *Revue* hereafter) followed this approach. *Revue* had been founded by Ribot, considered the father of experimental psychology in France, and proponent of the use of the pathological method. It almost never published photographs. As an exception, in 1904 the psychologist and Ribot’s mentee Georges Dumas published eight photographs of the experiments he had carried out at the Hôpital de Saint Anne (Figure 3).^36^ The experiments, which recalled those originally performed by Duchenne de Boulogne in 1962 and replicated by Charcot and Richer in 1881, consisted of the faradisation of facial muscles and nerves to investigate the pathology of the smile.^37^ Dumas, like Duchenne and Charcot before him, photographed the experiments and published the images together with the results.^38^ Photographs not only documented the experiments, but played a key role in them. The effects of the faradisation did not last for too long, and were available only to those who observed the experiment. Photography froze the expressions achieved by faradisation, allowing viewers to interpret those expressions, and therefore understanding the role of specific muscles and nerves in producing them. By making these photographs available in the press, the results of the experiment were open for discussion.

*Nouvelle* and *Revue*, therefore, integrated photographs into their arguments because they believed readers could interpret visual evidence for their own purposes. Once again, visualising the body was key in these journals. In contrast to this, photographs only served to represent the material settings and instruments in *L’Année*, whose articles relied on the data provided by tables and graphs.

## The Use of Artificial Lighting

Reading the signs of the body was not only a concern in medical journals. The popular press, and in particular the journals specialising in theatre, also sought to provide readers the means to interpret the inner life of the people portrayed in them.^39^*Le Théâtre*, launched in 1898, intended to make readers feel as if they had attended theatrical representations in Paris and beyond. To achieve this aim, the journal sought to represent with accuracy the artists’ performances, and the best means to capture a ‘history of gestures’ in ‘faithful plates’ was photography, as journalist Francisque Sarcey argued in the first issue.^40^ In this regard, *Le Théâtre* shared with scientific journals such as *Nouvelle* the belief that photography’s power to freeze the subject in an image would make their expressions readable.

**FIGURE 4.**
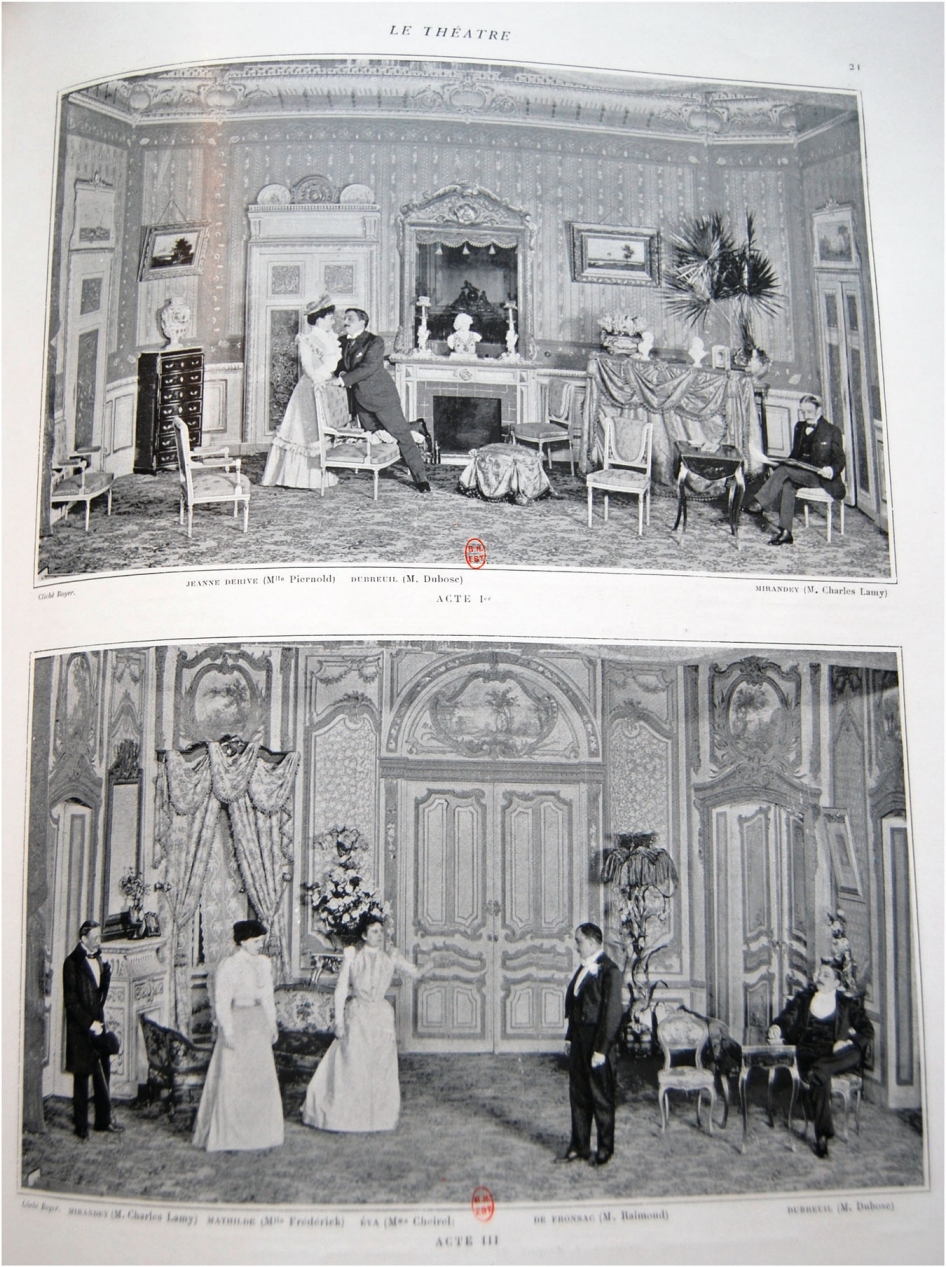
“Cliche Boyer. Acte 1. Jeanne Derive (Mme Piernold), Dubreuil (M. Dubose), Miranday (M. Charles Larry)”. Le Théâtre, 5 (1898), 21. Bibliotheque Nationale de France

Photographing actors onstage had remained a challenge until the development of the magnesium flash in the late 1880s.^41^ While artificial lighting was not standardised until the 1920s, some photographers achieved great success applying flash photography to theatre in the 1890s. One of them was Paul Boyer, from the Van Bosch studios in Paris. In 1892, Boyer had officially introduced his magnesium lamp to the Société Française de la Photographie, although he had been working on it for some years.^42^ Unlike the instantaneous flash, which produced a sudden bright light, Boyer’s lamp presented two main advantages. Firstly, it produced a continuous source of light, which allowed actors to get used to the light before the shutter was released. Secondly, several lamps could be linked to each other, so the light covered large stages.^43^ Boyer’s innovative photographs of the plays represented in the main Parisian theatres were described in newspapers such as *Le Figaro*, *Le Petit Parisienne* and *Le Journal*, and were published in *Le Photo-Programme* between 1895 and 1896. Boyer was also the photographer of most of the photographs in *Le Théâtre*, which proudly announced in its first number that readers would be able not only to see his ‘artworks’, but also to order enlargements of their favourite images (Figure 4).^44^

**FIGURE 5.**
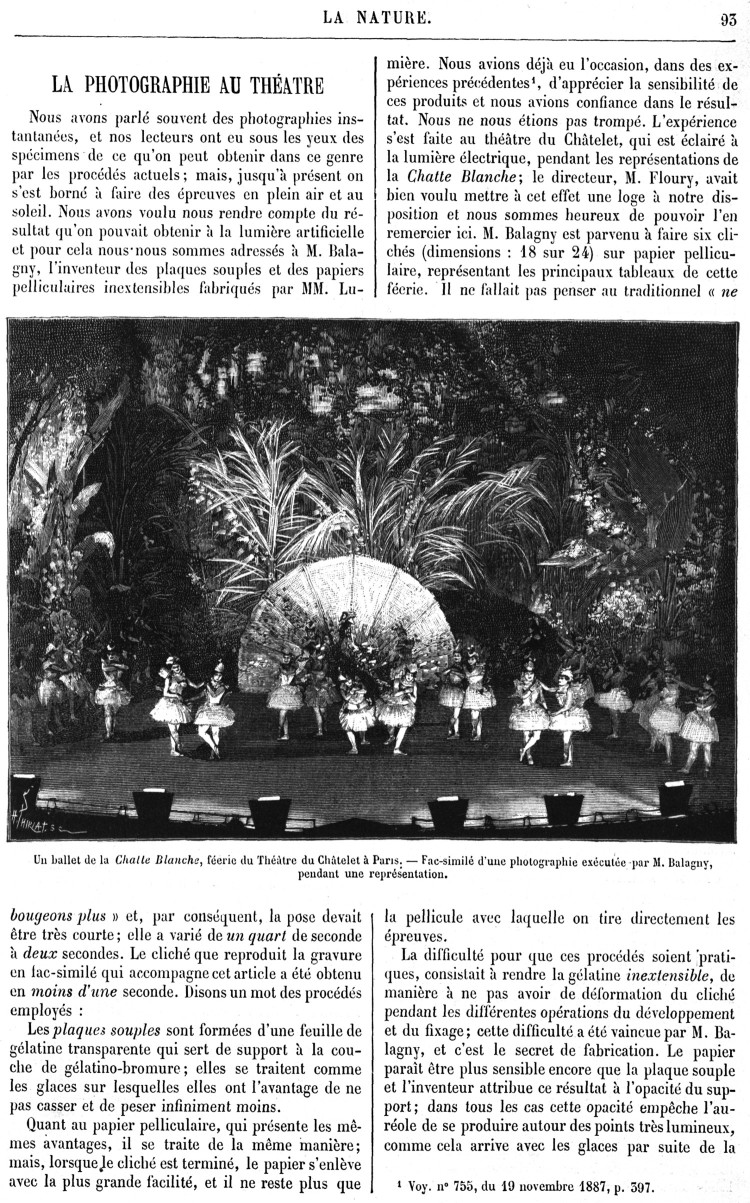
‘Un ballet de la Chatte Blanche, féérie du Théâtre du Chatelet à Paris’, La Nature, 16, n. 757–782 (1888): 93. Cnum-Conservatoire numérique des Arts et Métiers

While the popular press showed Boyer’s photographs and praised its quality, it did not comment on the problems involved in photographing actors, or on the qualities of the magnesium flash. This discussion happened in specialised journals such as the *Bulletin de la Société Française de la Photographie* and *Photo-Gazette*, targeted at professional photographers, as well as in scientific publications. *La Nature*, a popular science periodical founded by Gaston Tissandier, often disseminated the latest photographic procedures, such as colour and instantaneous photography.^45^ In 1888, some years before Boyer’s magnesium lamp, it had published G Mareschal’s ‘Photography in the theatre’, which explained how the photographer M Balagny had successfully captured live photographs of the representation of *Chatte Blanche* at the Châtelet theatre in Paris. In this case, two factors had come together: the availability of electrical lighting in the theatre, and Balagny’s production of soft plaques and inextensible films (Figure 5).^46^ Balagny’s method was different from Boyer’s, as he took advantage of the electrical lighting of the room, while Boyer and others worked on improving the use of artificial bright light that directly illuminated the subject. The innovations in this field were duly reported by *La Nature*, which took a great interest in the scientific explanations of artificial lighting.^47^

Mareschal’s article in *La Nature* included one of the six images that Balagny had taken at the Châtelet theatre. As usual in this journal, the photograph was reproduced as a facsimile of an engraving. As Belknap has argued, the fact that the image was consumed by the public as an engraving did not dismiss its authority, as it had been reproduced from a photograph.^48^ The bottom of the image allows us to see the stage’s footlights, which gave an indication of the technology used to illuminate the stage and therefore to produce the photograph. The engraving fulfilled the aims of the article. However, the quality of the reproduction prevented the reader from checking whether the photograph had captured the actors’ performance with enough detail. If readers wanted to see the actors’ gestures, they had to turn to *Le Théâtre, L’Illustration théâtrale* or *Le Photo-Programme*.

Popular and scientific journals tackled the application of artificial lighting to photography in the 1890s in different but complementary ways. While the scientific press was more concerned about the technological procedures that made the photography of actors possible, the popular press was interested in the resulting images. These two aspects of the same problem were very rarely addressed in the same space. An exception to this rule is *Photo-Journal*, a specialised photographic journal which combined theatrical reviews, discussions of technical aspects of photography (including artificial lighting) and good quality images, reproduced through the photo-mechanical process.^49^ Despite the different approaches to artificial lighting in these journals, they all had a common aim. Capturing the actors’ best performances was not only *Le Théâtre*’s goal*.* Contributors to *La Nature* also hoped that this new procedure would be able to make good images of theatrical representations, and the photographers writing in *Bulletin* also congratulated Boyer for his work in achieving this aim. While their approaches differed, scientific, popular and photographic journals shared the same concerns regarding the use of photography as a means to understanding the external signs of the body. In this regard, the discussions on artificial lighting and the images of theatrical representations were not very different from the debates in the medical press previously examined. In fact, Albert Londe, Head of the photographic service at the Salpêtrière and photographer of most of the photographs published in *Nouvelle*, also used the magnesium flash in his work.^50^ Together with photographic measurements and experiments, artificial lighting became a discussion point in the quest to determine the best methods for reading normal and pathological bodies.

## The Case for Chronophotography

Besides artificial lighting, the other photographic technology widely discussed in the French press was instantaneous photography, and particularly chronophotography. First invented by Eadweard Muybridge in 1877, chronophotography was the taking of several instantaneous images at regular (short) intervals of time to capture a subject in movement.^51^ The discussion of this new photographic application in the press shaped the terms of the debate.^52^ While it was mainly the medical and scientific press that engaged with this conversation, the ability to capture movement soon attracted the performing arts. Around 1893, Londe took several chronophotographs intended to help artists to represent nature and the human body in an accurate way. Among these images, Londe portrayed an acrobat kneeling on the trapeze.^53^ These chronophotographic images are very similar in their content to the photographs Londe took at the Parisian Hippodrome de l’Alma in the late 1880s, and later in 1891 as part of his experiments with artificial lighting.^54^ Londe was not the only one who applied chronophotography to capture performances. Georges Demenÿ, assistant of Marey at the Station Physiologique, also photographed ‘the cry and gesture of a famous actor’ in the context of his experiments on chronophotography.^55^ This series recalled Demenÿ’s famous ‘living portraits’, chronophotographic close-ups that showed in details the facial muscles involved in different utterances, such as ‘je t’aime’ and ‘vive la France’.^56^ Both Demenÿ’s and Londe’s projects linked the improvement of photographic technologies with a better or more accurate visual representation of the human body in movement. In this regard, the conversation around chronophotography was similar to the discussions on artificial lighting previously examined. As the following will show, both Demenÿ’s and Londe’s chronophotographic methods were also used in the medical press to strengthen approaches to the body in different disciplines, as discussed in the first two sections of this article.

Unsurprisingly, the two medical journals that engaged the most with chronophotography were *Nouvelle* and *L’Année*. In the case of *Nouvelle*, the link with chronophotography was evident. Londe had designed his own chronophotographic camera to help Paul Richer in his research on the physiology of movement, and opened an outdoors studio at the Salpêtrière specially adapted to their needs.^57^ In the spirit of sharing the visual documents produced at the Salpêtrière, *Nouvelle* published in 1895 an article by Richer on the shape of the body in movement, fully illustrated with Londe’s chronophotographs of a naked man lifting weights.^58^ In the same vein, Demenÿ wrote an article for *L’Année* in 1898 featuring Marey’s work at the Station Physiologique.^59^ In it, Demenÿ explained in detailed the technical characteristics and functioning of his chronophotographic camera, with the aim of insisting ‘on the role that [chronophotography] can play in physiology laboratories’.^60^

Both Marey and Londe’s chronophotographic methods were intended to enhance visual perception by rendering visible what escaped the human eye. However, the visualisation of movement followed different principles in each case: while Marey intended to measure the direction and speed of movement, Londe wanted to reveal the external changes in the muscles produced by movement.^61^ These two purposes required different cameras. Marey constructed a single lens camera that analysed movement by capturing a succession of photographic images, and synthesised it through its filmic projection. The figures were recorded on the same plate, which often produced overlaps. Marey and Demenÿ’s approach corresponded to the interests of experimental physiology, as they used chronophotography to translate the visible world into data that would later be analysed.^62^

In contrast, Londe was interested in the external shape of the body. While the direction of the movement was important in cases of pathological locomotion, in the studies of normal physiology the focus was on the visible changes experienced by the muscles. To this end, Londe built a camera with twelve shutters that were shot successively on the same plate.^63^ The final image consisted of three rows of four columns, each showing an instant of the movement with no overlapping whatsoever. Unlike Marey, Londe sought to highlight the body of the subject, and photographed the subjects naked against a grey background. Even if Richer's project was on physiology and not pathology, this method followed the principles of the anatomo-clinical medicine. As with the indoor studio Londe had set up at the Salpêtrière, his chronophotographic camera, outdoor stage and plates were designed to enhance the visual observation of the body.

The publication of Demenÿ’s article in *L’Année* was the journal’s stance on how to use chronophotography. Binet had written a review in 1895 praising Richer’s *Physiologie Artistique*, recognising the relevance of the study to psychology.^64^ He mentioned the chronophotographs that illustrated the book, but only to recall Marey’s and Demenÿ’s work, without even mentioning Londe. On the other hand, *Nouvelle* did not show Marey’s work, concentrating on Londe’s approach to chronophotography. The different ways in which *L’Année* and *Nouvelle* featured this photographic invention provide a good example of how particular photographic technologies identified with the scientific aims and methods of each publication. The only outlet that frequently published works by both authors was *La Nature*, which took a broad approach to science and never identified with a single school of thought. Journals, therefore, became a privileged site not only to discuss the technical details and potential uses of chronophotography, but also the different disciplinary approaches to the visualisation of the body in movement.

## Conclusion

The survey of the role of photography in major journals in French psychology and physiology (*Nouvelle*, *Archives* and *L’Année*) and others such as *Le Théâtre* and *La Nature* have demonstrated that photographic reproductions in the press became a key site where ideas regarding the body, its external signs and their interpretation were discussed. Most of the contributors examined in this article defended the accuracy of photographs in representing fleeting expressions, but not all of them. As we have seen, Binet maintained that photographs were not able to convey natural expressions. This disagreement reflected tensions between disciplines and schools of thought, and projected different images of science. While *L’Année* represented psychology as an activity involving physiological experiments and instruments, *Nouvelle*, *Archives* and *Revue* reinforced visual observation through the display of bodily pathologies in the clinic. Both images of science were commented on in *La Nature*, but only one penetrated into popular journals. *Le Théâtre*’s belief that photographs allowed the truthful representation of actors’ performances, and that the publication of these images in journals would enable a history of gestures, connected it with the aims and practices of *Nouvelle*.

Photographs printed in the press became objects of a particular kind, different from the prints stored in hospitals and photographic studios. In this move, photographs brought together and drew apart different fields. Medical journals reaffirmed their own disciplinary identities through the role they granted to photographs. But, as Demenÿ’s chronophotographs of an actor show, photographs were mixed material that circulated across spaces: the same image could be published in a psychology journal and also contribute to theatre studies. Photographs did not belong to either theatre or science, but was what connected both fields.

## Notes

1. See Callen, *The Spectacular Body* and Wallis, *Investigating the Body*.

2. Duchenne, *Mécanisme*; Darwin, *The Expression*. See Delaporte, *Anatomie*.

3. Kalunszynski, “Alphonse Bertillon.”

4. Goetz, “Jean Martin Charcot,” 203–212.

5. Gordon, “From Charcot,” Gordon, *Why the French*, also Gilman, “The Image,” 345–436; Justice-Mallow, “Charcot,” 133–138. See also Didi-Huberman, *Invention of Hysteria*; Hunter, *The Face* and Hustvedt, *Medical Muses*.

6. Belknap, *From a Photograph*, 6. There was also a surge in the popular press, see Gervais, *La Fabrique*.

7. On science and photography, see Tucker, *Nature Exposed*; Wilder, *Photography* and Daston & Galison, *Objectivity*.

8. Belknap, *From a Photograph*.

9. Elizabeth Edwards has argued that the materiality of the photographic prints contributed to their consideration as scientific objects in “Material Beings,” 71.

10. Belknap, *From a Photograph*, 6.

11. Ibid.; Edwards and Hart, *Photographs, Objects, Histories*.

12. Richer, Tourette, and Londe, “Avertissement.”

13. Ibid.

14. Ibid.

15. Ibid.

16. Faure, “La photographie,” 104–124.

17. Bourneville, “Idiotie profonde,” 38–55.

18. The text mentions the years 1890, 1892, 1894, 1895, 1896, 1897, 1899 and 1902. Ibid., 41, 43, 44, 45, 47, 48, 49 and 51.

19. Ibid., 44.

20. See for instance the series of articles on a patient suffering from idiocy and myxomatosis who had recently died. Bourneville, “Fin de l’histoire,” 97–119.

21. Brown, “Racialising the Virile Body,” 631.

22. Pinney, *Photography and Anthropology*, 29. See also Brown, “Racialising the Virile Body.”

23. Bourneville, “Idiotie profonde,” 38–55.

24. Something similar happened in the examples examined by Mark Jackson in “Images of Deviance,” 319–337.

25. On the history of *L’Année*, see Vermès, “L’Année Psychologique,” 113–129.

26. Binet, “Recherches complémentaires,” 375–402.

27. Ibid, 382.

28. Ibid, 382.

29. See Plas, “Psychology,” 91–107, Nicolas and Charvillat, “Introducing Psychology,” 143–164, and Nicolas, Segui and Lefrand, “Les premières revues,” 71–110.

30. Binet’s relationship with images has been examined by Mireille Berton, “Alfred Binet,” 197–202.

31. Binet, “Causerie pédagogique,” 405–431.

32. Ibid., 428.

33. Ibid. 428

34. Binet cited here the work of Dr Badaloni, who had demonstrated a correlation between bad posture, where the table compresses the chest of the child, and the decrease of breathing. Ibid. 425–428.

35. Evans, “Psychological Instruments,” 113–129.

36. Dumas, “Le sourire,” 1–23.

37. Duchenne (de Boulogne), *Mécanisme*, Charcot and Richer “Contribution,” 32–75.

38. I have discussed these photographs in Pichel, “Die Psychologie.”

39. The next two sections will focus on photography, but similar analyses have been carried out by Mireille Berton in relation to cinema and psychology (Berton, *Le Corps*) and by Garcin Marrou regarding theatre and psychology (Garcin Marrou, “André de Lorde”).

40. Sarcey, “Le théâtre instantané”

41. While the first experiments with magnesium started in the 1860s with Alfred Brothers and Charles Piazzi, the magnesium flash powders were not introduced until the late 1880s. Pritchard, “Artificial Lighting.” For a cultural history of flash, see Kate Flint, *Flash!.*

42. S.G.D.G., “Lampe-éclaire Boyer,” 160–162.

43. Ibid., 160.

44. *Le Théâtre*, n. 1, January 1898.

45. On *La Nature*, see Manuel Chemineau, *Fortunes*.

46. Mareschal, "La Photographie", 93.

47. for instance Londe, “Photographie instantanée,” 343–346.

48. Belknap, *From a Photograph*.

49. Peutat, “Derrière le Rideau,” 97–99.

50. Londe, “Contribution à l’étude,” 102–103.

51. On Muybridge, see Braun, *Muybridge*.

52. Belknap, *From a Photograph*, 152.

53. Londe, *Album de chronophotographies*, plate XV.

54. Londe’s photographs of acrobats at the Hippodrome de l’alma are available online at the Musée d’Orsay, “Album composé dans l’entourage d’Albert Londe” http://www.musee-orsay.fr/en/collections/index-of-works/notice.html?no_cache=1&ens=1&nnumid=172164&retouroeuvre=%252Fen%252Fcollections%252Findex-of-works%252Fnotice.html%253Fno_cache%253D1%2526zsz%253D5%2526lnum%253D8. Accessed on February 1, 2018.

55. Demenÿ, “Les appareils chronophotographiques,” 367.

56. Demenÿ, “Le portrait,” 1–4.

57. Londe, “La photochronographie,” 370–374.

58. Richer, “De la forme,” 122–135.

59. Demenÿ, “Les appareils chronophotographiques,” 347–368.

60. Ibid., 368.

61. See Braun and Whitcombe, “Marey, Muybridge,” 218.

62. Braun, *Picturing Time*.

63. Londe, “La photochronographie,” 370–374.

64. Binet, “P. Richer,” 731–740.
